# Supplementary material for: Comparison of the Fecal Bacteriome of HIV-Positive and HIV-Negative Older Adults
Source: Biomedicines. 2023 Aug 19;11(8):2305. doi: 10.3390/biomedicines11082305 (PMC10452058; doi:10.3390/biomedicines11082305)
Supplement: Supplementary file 1 [file biomedicines-11-02305-s001.zip › Table S1 Biomedicines.pdf]

Table S1. Alpha and beta diversity in the fecal samples of the patients of the PWH groups according to other variables assessed in this study.

**Variables**	**Samples (n)**	**Shannon***	**Bray-Curtis****	**Jaccard****
**Depression**				
No-drepression	17	3.59 (3.44-3.69)	-	-
Depression	7	3.29 (3.23 3.66)	-	-
p-values	-	0.26	0.3	0.39
**Nadir**				
Nadir≥200	8	3.66 (3.48-3.79)	-	-
Nadir<200	16	3.51 (3.32-3.62)	-	-
p-values	-	0.32	0.65	0.36
**Walking speed**				
**<0.8 m/sg**	3	3.59 (3.55-3.6)	-	-
**0.8 – 1.2 m/sg**	3	3.23 (3.19-3.26)	-	-
**>1.2 m/sg**	18	3.62 (3.41-3.71)	-	-
p-values	-	0.06	0.32	0.19
**SPPB**				
<=6 points	3	3.59 (3.41-3.61)	-	-
7-9 points	3	3.51 (3.32-3.68)	-	-
>=10 points	18	3.66 (3.4-3.74)	-	-
p-values	-	0.67	0.24	0.19
**Polypharmacy**				
No polypharmacy	12	3.52 (3.32-3.65)	-	-
Yes polypharmacy	12	3.56 (3.44-3.7)	-	-
p-values	-	0.89	0.31	0.35

*Wilcoxon-Mann-Whitney test for variables with 2 factors (depression, Nadir, polypharmacy) and Kruskal-Wallis test for variables with 3 factors (walking speed, SPPB).
** PERMANOVA test with 999 permutations.
